# From Yield to Flavor: The Role of Lipid Coatings in Beef Aging

**DOI:** 10.1111/1750-3841.71240

**Published:** 2026-06-29

**Authors:** Jonatã Henrique Rezende‐de‐Souza, Kathelen Lethicia Cavalheri Rodrigues Jacinto, Isaac de Lima Vieira, Gabriela Lima de Oliveira, Isabela Benfica de Barros, Flavio Andre Bolini Cardello, Dyana Carla Lima Hargreaves Noguera, Vanessa Cristina Francisco, Renata Tieko Nassu, Helena Maria Andre Bolini, Sérgio Bertelli Pflanzer

**Affiliations:** ^1^ Department of Food Engineering and Technology School of Food Engineering University of Campinas Campinas São Paulo Brazil; ^2^ Embrapa Pecuária Sudeste São Carlos São Paulo Brazil

**Keywords:** butter‐aged, CATA, internal preference map, loin, meat flavor, volatile compounds

## Abstract

Lipid‐coated aging has been proposed as an alternative to enhance flavor, particularly using butter as a coating material (butter‐aged beef). Thus, this study compared the wet‐aged and beef‐aged with different lipid‐based coatings: milk butter (butter‐aged), cocoa butter (cocoa‐aged), pork lard (lard‐aged), and beef tallow (tallow‐aged), all after 8 days of aging. Beef portions with lipid coatings developed cracks, particularly those coated with milk butter (16.7%), cocoa butter (100%), and beef tallow (100%), causing meat surface darkening. In lipid‐coated samples, total weight loss (14.3%–16.6%) and yield (83.4%–85.7%) were not affected by treatments. Water activity was higher in initial samples (0.9909) compared to lipid‐coated samples (0.9884–0.9890) (*p* < 0.05). The aging process, with or without lipid coating, increased lightness and decreased redness, yellowness, and chroma compared to initial samples. Display storage time decreased L*, a*, and chroma values and increased hue angle, particularly after 6 days compared to Day 0 (*p* < 0.05). Sensory evaluation revealed significant differences among treatments for aroma, flavor, and overall impression. Butter‐ and lard‐aged samples showed slightly higher aroma and flavor scores compared to tallow‐aged samples. Lipid‐coated aging influenced the volatile profile. Principal component analysis revealed that wet‐, butter‐, cocoa‐, and lard‐aged samples were more associated with positive descriptors, which were positively associated with overall impression scores, whereas tallow‐aged samples were associated with less desirable descriptors such as “bitter” and “rancid.” In conclusion, lipid coatings promoted sensory modifications, supported by differences in volatile compounds, but did not improve consumer acceptance compared to wet‐aged samples.

**Practical Applications:**

Lipid‐coated beef aging represents an alternative approach to explore modifications in aroma‐related attributes of aged meat. Under the conditions evaluated in this study, this technique promoted significant changes in aroma, flavor, and overall impression, which were supported by differences in volatile compound profiles. However, these changes did not translate into substantial improvements in overall consumer acceptance compared to conventional wet aging. This technique may be of interest for exploratory or niche applications focused on flavor differentiation or product innovation. However, the practical advantages of lipid‐coated aging remain dependent on processing conditions and should be further investigated.

## 1 Introduction

The aging process of meat is a natural endogenous process based on the proteolytic action of the calpain enzyme complex on the structural proteins, leading to sarcomere degradation and resulting in increased tenderness compared to non‐aged meat (Bhat et al. 2018; Dransfield 1993). Currently, two well‐established aging methods are employed: dry‐ and wet‐aging, with the latter being the most widely used. In wet‐aging, meat is vacuum‐packed in low‐gas‐permeability packaging and stored at low temperatures (−1°C–4°C) for specific periods. Despite its practicality and high process yield (Zhang et al. 2022), wet aging can lead to meat color changes (dark purplish‐red or purplish‐pink color) caused by the low oxygen pressure inside the packaging, which can compromise consumer acceptance (King et al. 2023). Furthermore, from a sustainability perspective, concerns have been raised regarding the environmental impact of plastic packaging waste (Gil and Rudy 2023), such as in vacuum‐aging systems.

In contrast, dry aging involves storing meat without packaging in refrigerated chambers with controlled temperature, relative humidity, and air circulation for several weeks (Dashdorj et al. 2016; Rezende‐de‐Souza et al. 2021). The absence of packaging enhances the meat's sensory characteristics, improving its palatability. However, exposure to air leads to surface dehydration, which can reduce process yield by up to 50%, thereby increasing the final cost for consumers (Bernardo et al. 2020; Bernardo, Silva, et al. 2020). To mitigate weight loss, reduce lipid oxidation, extend shelf life, and improve sensory attributes during aging, studies have proposed the application of polysaccharide‐based coatings, including biopolymers and edible biofilms.

Inspired by the principles of polysaccharide coatings, gourmet meat restaurants and small‐scale producers have introduced lipid‐based coatings prior to the aging process, as reported in social media and non‐scientific sources. In addition to improving process yield, these producers aimed to enhance the sensory characteristics of the final product. Rezende‐de‐Souza et al. (2021) reported that among 37 Brazilian dry‐aged meat producers, 24.3% used some form of lipid coating before aging, marking the introduction of butter‐aged in Brazil. However, during this period, no scientific studies had evaluated the production parameters and final quality of meat aged with lipid coatings, with available information being limited to empirical observations shared among producers and online sources. The main ingredients used to coat the meat, as seen on various social media platforms, include milk butter, pork lard, beef tallow, chocolate, and hazelnut cream, among other animal or vegetable lipids, with or without spices and seasonings. Thus, in addition to enhancing the flavor, the cost of these ingredients was also a factor in their selection. Subsequently, a pioneering study assessed the final quality of beef‐aged for 28 days with lipid coatings, specifically using milk butter and pork lard (Rezende‐de‐Souza et al. 2024). Given this context, in the present study, we aimed to investigate the aging of beef using different lipid sources as coating materials for a short aging period, comparing them with initial samples and wet‐aged meat to characterize their volatile profile and assess their impact on sensory quality.

## 2 Materials and Methods

This study was approved by the Ethics Committee of the State University of Campinas, under No. 15232819.8.0000.5404.

### 2.1 Raw Materials

Twelve boneless loin cuts (∼30 cm) from the *Longissimus thoracis* et *lumborum* (8th–12th thoracic vertebra) were purchased from a local retailer in Campinas, São Paulo, Brazil. All samples were purchased on the same day from the same retail location and selected based on consistent labeling information, including brand, commercial lot, production date, cut size, and breed certification. All samples originated from Zebu cattle and were vacuum‐aged for 30 days prior to the experimental treatments. The 30‐day vacuum aging period corresponded to the commercial aging time indicated on the product labeling at the time of acquisition. The end of this initial aging period (30 days) was designated as Day 0 for the laboratory aging procedures. Coating materials included commercial milk butter (Milkpar, Brazil), refined pork lard (Seara, Brazil), beef tallow (Friboi, Brazil), and deodorized cocoa butter (Cargill, Brazil).

### 2.2 Samples Preparation and Aging Process

Subcutaneous fat was removed to enhance lipid coating adhesion, as recommended by Rezende‐de‐Souza et al. (2024). Each loin was divided into three equal portions (∼10 cm each), totaling 36 portions, randomly assigned to six treatments: initial samples, wet‐aged, butter‐aged, cocoa‐aged, lard‐aged, and tallow‐aged (*n* = 6).

Initial samples were used for baseline characterization. From the cranial region of each portion, samples were taken for water activity (one steak, 0.5 cm thick), instrumental color and volatile compounds (two steaks, 1.5 cm thick), and instrumental tenderness and sensory evaluation (two steaks, 2.5 cm thick). Water activity and instrumental color analyses were performed on the processing day; remaining samples were vacuum‐packed and frozen at −18°C until analysis.

For the wet‐aged treatment, six portions were weighed, vacuum‐packed, and stored in an aging chamber at 1°C for 8 days. The packaging (Cryovac BB 2620, Cryovac Brasil Ltda) had a thickness of 50 µm, an oxygen permeability of 20 cm^3^/m^2^·24 h at 23°C and 75% RH, and a maximum carbon dioxide permeability of 100 cm^3^/m^2^·24 h at 23°C and 75% RH.

For all lipid‐coated treatments, each portion was tied with culinary string on sanitized butcher hooks (0.2% peracetic acid) and suspended in an aging chamber (1°C, 2 m/s air velocity, 75% RH) for 24 h to allow surface drying and improve lipid adhesion. After drying, coating lipids were melted at 50°C and cooled to 18°C (Rezende‐de‐Souza et al. 2024), and each sample was immersed once in its respective lipid, for approximately 1 min. Samples were refrigerated in the chamber at 1°C for 2 h to solidify the coating and then randomly distributed in the aging chamber for 7 days under the same suspension conditions of samples and aging chamber configuration.

At the end of the aging process, lipid coating thickness was measured with a caliper. For all aged samples, packaging or coatings were removed, any visual changes in the samples were observed and recorded, and portions were weighed to calculate yield. Water activity, color, tenderness, volatile compounds, and sensory analyses were performed using the same procedures as for initial samples.

### 2.3 Process Yield

Weight measurements were used to determine adherence to lipid coating and to calculate the total loss due to dehydration and the total yield (Equations (1), (2), (3)). For Equations (2) and (3), the fat mass was weighed together with the meat mass, before and after the aging process. This is because in our preliminary studies, we identified that removing the fat after aging left traces of fat in the meat, which affected the final weight. Therefore, the meat–fat combination was weighed, and then the weight of the fat was deducted to obtain the individual mass of the meat.

(1) $$\mathrm{Adhered}\;\mathrm{lipid}\;\mathrm{coating}\;\left(\%\right)=\frac{\mathrm{Adhered}\;\mathrm{lipi}d_{\left(g\right)}\times 100}{\mathrm{Weight}\;\mathrm{of}\;\mathrm{bee}f_{\left(g\right)}}$$

(2)

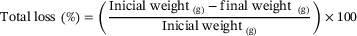

(3) $$\mathrm{Total}\;\mathrm{yield}\;\left(\%\right)=100-\mathrm{total}\;\mathrm{loss}$$

### 2.4 Water Activity

Water activity was measured on surface steak samples. Strips 0.5 cm thick were collected from the external surface of the meat that had been in direct contact with the lipid coating and extended inward toward the interior of the muscle (cranial direction of the original carcass orientation). Measurements were performed using an Aqualab 4TE water activity meter (Decagon, Brazil). Two measurements were taken per sample, and the mean value was used for statistical analysis.

### 2.5 Instrumental Color

For instrumental color assessment, steaks were placed in expanded polystyrene trays, wrapped in polyvinyl chloride film, and stored in a refrigeration chamber at 2°C without internal lighting. Color measurements were performed using a MiniScan MSEZ 1314 portable colorimeter (HunterLab, Reston, VA), calibrated with black and white plates. Measurements were taken in three different regions of the steak, avoiding coarse connective tissue. The device was set with illuminant D65, a viewing angle of 8°, and a standard observer of 10°. Lightness (L*), redness (a*), and yellowness (b*) values were obtained, and chroma and hue angle were calculated using equations described by King et al. (2023). Initially, samples were allowed to oxygenate for 1 h before the first measurement (Day 0). Subsequent color readings were conducted every 48 h, corresponding to days 2, 4, and 6 of storage.

### 2.6 Instrumental Tenderness and Sensory Analysis

The procedures followed the methodology outlined by the American Meat Science Association (AMSA 2015). After thawing, a 2.54 cm raw sample was weighed and placed on a baking tray with a rack. A thermocouple was inserted into the sample's center, and it was cooked in a conventional oven preheated to 175°C (Fritomaq 90 × 90, São Paulo, Brazil). When the sample reached 45°C internally, it was flipped and fully cooked until it reached 70°C internally. After cooking, the samples were weighed while still hot for cooking loss calculation.

Immediately after cooking and weighing, the samples were sampled for instrumental texture analysis using the Slice Shear Force method (AMSA 2015). A 1 cm thick and 5‐cm long slice was taken from the lateral end of the sample. A second cut was made toward the medial side, parallel and 5 cm from the first cut, to obtain a 5 cm section. A third cut was made through the length axis using a double‐bladed knife, spaced 1 cm apart, at a 45° angle to the *Longissimus* axis and parallel to the muscle fibers. The slice was sheared with a straight blade of 1.016 mm thickness using a TA‐XT texture analyzer (Stable Micro Systems, Surrey, UK), set to a speed of 3.7 mm/s.

From the remaining warm sample, 2 × 2 cm portions were cut, placed in labeled glass jars, and stored at 40°C (Gomes et al. 2014). The sensory panel consisted of 56 untrained consumers who reported consuming beef at least once per week and were recruited on a voluntary basis; all participants provided informed consent prior to the sensory evaluation. No prior selection criteria were applied regarding education level or other sociodemographic characteristics. Among participants, 51.7% identified as female and 48.3% as male. Age distribution was similar between genders; therefore, age was described for the overall panel: most participants were between 18 and 29 years old (84.5%), followed by those aged 30–44 years (13.8%) and 45–59 years (1.7%). Each panelist received an individual sample portion along with evaluation sheets. Acceptance tests were conducted using a 7‐point hedonic scale: 1, strongly dislike; 2, dislike very much; 3, dislike; 4, neither like nor dislike; 5, like; 6, like very much; and 7, strongly like (AMSA 2015, with modifications). The Check‐All‐That‐Apply (CATA) method was also applied, identifying descriptor terms related to taste and flavor attributes: acidic, bitter, metallic, rancid, salty, bloody, normal meat flavor, umami, roasted, cooked, toasted, cocoa butter, liver, milk butter, pork lard, and beef tallow.

### 2.7 Volatile Compounds

The procedure followed the protocol described by Rezende‐de‐Souza et al. (2024), identifying compounds qualitatively. Therefore, results were interpreted as relative comparisons rather than absolute quantification. Samples were grilled in a preheated Ford 9 electric oven (NKS, São Paulo, Brazil) at 180°C for 10 min until reaching an internal temperature of 70°C, monitored by a thermometer. After cooking, samples were placed in a plastic bag, cooled in an ice bath for 10 min, and crushed in a Viva RI1364/06 food processor (Walita, São Paulo, Brazil). For volatile compound extraction, 1 g of the cooked, ground sample was mixed with 5 mL of saline solution containing 0.01% BHT, sealed with a PTFE/silicone septum, and stirred at 30°C. A CAR/PDMS SPME fiber was used for 5 min of equilibration and 65 min of extraction. Analysis was performed using a QP‐2010 gas chromatograph (Shimadzu, Kyoto, Japan) with a quadrupole mass spectrometer. Thermal desorption at 250°C was performed in splitless mode for 1 min, with 12 min for complete desorption. A fiber blank was used between each procedure to prevent carryover. Volatile compounds were separated on a DB‐5 ms MS column (J&W Scientific, CA, USA). The oven temperature started at 40°C, increased to 180°C at 4°C/min, then to 280°C at 10°C/min, holding for 5.3 min. Helium was used as the carrier gas at 1 mL/min. The detector used 70 eV ionization energy, 300°C interface, 250°C ion source, and scanned *m/z* 35–350. Compound identification was done using the NIST GC‐MS library (Thomas 2002).

### 2.8 Statistical Analysis

Yield, water activity, cooking loss, and instrumental tenderness were analyzed using linear mixed‐effects models, considering treatment as a fixed effect and animal and muscle position as random effects.

For color analysis, potential outliers were identified by comparing replicates within each measurement using the median absolute deviation (MAD), adopting a rejection threshold of 3 (Leys et al. 2013). Subsequently, color parameters were analyzed using linear mixed‐effects models, including treatment, storage time, and their interaction as fixed effects. To account for repeated measurements over time, sample (defined as the combination of animal and muscle position) was included as a random effect, with a random slope for time to allow individual trajectories. Sensory data obtained using a structured hedonic scale were analyzed using linear mixed‐effects models, including treatment as a fixed effect and panelist (consumer), animal, and muscle position as random effects.

For all models (yield, water activity, cooking loss, instrumental tenderness, instrumental color, and sensory attributes), when significant effects were detected, multiple comparisons among means were performed using Tukey's test at a 5% significance level. These analyses were performed using RStudio Software (version 4.5.2).

CATA sensory data were analyzed using correspondence analysis (CA). In complement, the internal preference map (IPM) was developed, derived from principal coordinates analysis (PCoA) to explore the relationships between sensory descriptors, treatments, and overall acceptability. Volatile compounds were initially assessed qualitatively; subsequently, relative peak areas were used for exploratory principal component analysis (PCA) by semi‐quantitative approach, without normalization or absolute quantification. CA, IPM, and PCA analyses were performed using XLSTAT (version 2025.1).

Pearson correlation analysis was performed using the mean sensory scores obtained for each steak sample, allowing the sensory and physicochemical datasets to share the same experimental unit (steak); this approach was adopted to avoid pseudo‐replication due to the different number of observations between sensory and instrumental analyses. The color data were analyzed only for Day 0 on the display. Pearson's correlation was performed using RStudio Software (version 4.5.2).

## 3 Results

Loin cuts coated with lipid materials showed no signs of contamination or excessive exudation. However, cracks in the coating layer were observed in some treatments, particularly butter‐aged, cocoa‐aged, and tallow‐aged samples (16.7%, 100%, and 100%, respectively). Due to the variability in crack size and shape, quantitative measurements were not feasible. Therefore, crack occurrence was expressed as the percentage of samples exhibiting visible cracks within each treatment. No cracks were observed in lard‐aged samples, which may be associated with differences in rheological properties and crystallization behavior compared with the other lipid sources evaluated. Coating thickness was similar across treatments, averaging around 10 mm. After removing the coatings, all samples from all treatments displayed a brown surface color (data not shown in table).

Cocoa butter showed the highest adhesion to the meat compared to milk butter (*p* < 0.05), while the other coatings did not differ significantly (Table 1). Total weight loss and total yield were not significantly affected by the type of lipid coating used during beef aging (Table 1).

**TABLE 1:** Yield of beef‐aged with lipid coating.

Attribute (%)	Butter‐aged^a^	Cocoa‐aged^a^	Lard‐aged^a^	Tallow‐aged^a^	SEM	*p*‐value
Lipid adhesion^b^	31.2 b	36.2 a	33.2 ab	35.1 ab	1.23	< 0.05
Total loss^c^	16.3	15.1	16.6	14.3	0.68	0.06
Total yield^d^	83.7	84.9	83.4	85.7	0.68	0.06

*Note*: Means equal letters in the same row are not significantly different (*p* < 0.05).

Abbreviation: SEM, standard error of the mean.

^a^ Samples subjected to 30 days of prior commercial vacuum aging, followed by the experimental aging process, with or without lipid coating.

^b^ Liphid adhesion = (Adhered lipid × 100)/weight of meat.

^c^ Total loss = (Initial weight − final weight)/(initial weight) × 100.

^d^ Total yield = 100 − total loss.

Initial samples had the highest water activity compared to all aged samples with lipid coatings (*p* < 0.05), but without differing from wet‐aged samples (*p* > 0.05). Regarding the samples aged with lipid coatings, only butter‐aged did not differ significantly from wet‐aged samples (Table 2).

**TABLE 2:** Water activity of beef‐aged with lipid coating.

Attribute	Initial samples^a^	Wet‐aged^b^	Butter‐aged^b^	Cocoa‐aged^b^	Lard‐aged^b^	Tallow‐aged^b^	SEM	*p*‐value
Water activity	0.9909 a	0.9903 ab	0.9890 bc	0.9885 c	0.9877 c	0.9884 c	0.0004	< 0.001

*Note*: Means equal letters in the same row are not significantly different (*p* < 0.05).

Abbreviation: SEM, standard error of the mean.

^a^ Samples subjected only to 30 days of prior commercial vacuum aging.

^b^ Samples subjected to 30 days of prior commercial vacuum aging, followed by the experimental aging process, with or without lipid coating.

No significant interaction between treatment and storage time was observed for any color parameter (Table 3). Regarding treatment effects, lard‐aged samples exhibited higher lightness values (42.6), not differing significantly only from cocoa‐aged samples (41.2). In contrast, initial samples showed the lowest lightness (39.3), with values comparable to butter‐ and tallow‐aged samples. Initial samples presented significantly higher redness, yellowness, and chroma compared to all aged treatments. Treatment had no significant effect on hue angle, with values ranging from 39.2 to 42.6 (Table 3).

**TABLE 3:** Color stability during storage time of beef‐aged with lipid coating.

Attribute	Treatment (T)						Storage time (D)				SEM	*p*‐value		
	Initial samples^a^	Wet‐aged^b^	Butter‐aged^b^	Cocoa‐aged^b^	Lard‐aged^b^	Tallow‐aged^b^	Day 0	Day 2	Day 4	Day 6		T	D	T × D
L*	39.3 c	40.9 b	40.6 bc	41.2 ab	42.6 a	39.7 bc	43.4 a	40.7 b	39.5 bc	39.3 c	2.38	< 0.001	< 0.001	0.84
a*	21.9 a	16.9 b	17.3 b	17.1 b	16.6 b	17.0 b	20.6 a	20.0 a	16.6 b	14.0 c	2.36	< 0.001	< 0.001	0.23
b*	17.4 a	14.6 b	15.1 b	14.6 b	14.0 b	15.1 b	15.30	15.8	14.8	14.6	1.78	< 0.001	0.07	0.26
Chroma	27.9 a	22.5 b	23.0 b	22.5 b	22.0 b	22.8 b	25.7 a	25.5 a	22.3 b	20.3 b	2.81	< 0.001	< 0.001	0.15
Hue angule	39.2	42.6	41.8	41.0	40.2	41.9	36.5 b	38.6 b	42.1 a	47.4 a	1.04	0.32	< 0.001	0.94

*Note*: Means equal letters in the same row are not significantly different (*p* < 0.05).

Abbreviation: SEM, standard error of the mean.

^a^ Samples subjected only to 30 days of prior commercial vacuum aging.

^b^ Samples subjected to 30 days of prior commercial vacuum aging, followed by the experimental aging process, with or without lipid coating.

Storage time significantly affected most color parameters (Table 3). Lightness decreased over time, with lower values observed on Day 6 (39.3) compared to days 2 (40.7) and 0 (43.4). Redness progressively declined from days 0 to 6, with higher values on days 0 and 2 (20.6–20.2) compared to days 4 (16.6) and 6 (14.0). Similarly, chroma decreased over time (*p* < 0.05), with higher values at days 0 and 2 (25.7–25.5), followed by a reduction at days 4 and 6 (22.3–20.3). The hue angle increased significantly throughout storage, ranging from 36.5–38.6 on days 0 and 2 to 42.1–47.4 on days 4 and 6. In contrast, yellowness was not affected by storage time (*p* > 0.05), remaining relatively stable (14.6–15.8).

Cooking loss was significantly lower in cocoa and tallow‐aged samples compared to wet‐aged samples, whereas initial samples and butter‐ and lard‐aged samples did not differ from the other aging processes (Table 4). Instrumental tenderness ranged from 13.9 N to 17.6 N, with no significant variation among treatments, which aligned with sensory tenderness ratings, as they were also unaffected by treatment and ranged from 5.18 to 5.54 (Table 4). Juiciness ranged from 4.87 to 5.40 and did not differ among the six treatments evaluated in this study (*p* < 0.05). In contrast, significant differences were observed for aroma, flavor, and overall impression among treatments (*p* < 0.05). Aroma was significantly higher in the butter‐ and lard‐aged samples compared to the tallow‐aged samples, while the remaining treatments did not differ from each other. Flavor and overall impression followed a similar pattern; flavor scores were higher for lard‐aged samples (4.98), and overall impression was higher for wet‐aged samples (5.23), both compared to tallow‐aged samples (4.39 and 4.63, respectively).

**TABLE 4:** Cooking loss, instrumental texture and sensory profile of beef‐aged with lipid coating.

Attribute	Initial samples^a^ ^A^	Wet‐aged^b^	Butter‐aged^b^	Cocoa‐aged^b^	Lard‐aged^b^	Tallow‐aged^b^	SEM	*p*‐value
Cooking loss, %	22.4 ab	24.1 a	20.5 ab	18.7 b	19.6 ab	18.3 b	1.36	< 0.01
Slice shear force, N	17.6	17.3	16.7	14.7	15.1	13.9	3.12	0.20
Tenderness^c^	5.51	5.46	5.33	5.30	5.54	5.18	0.16	0.42
Aroma^c^	5.09 ab	4.98 ab	5.16 a	4.98ab	5.19a	4.61 b	0.16	< 0.05
Flavor^c^	4.93ab	4.91ab	4.93ab	4.79ab	4.98 a	4.39 b	0.17	< 0.05
Juiciness^c^	5.40	5.17	5.01	4.87	4.99	4.89	0.19	0.11
Overall impression^c^	5.14ab	5.23 a	5.14ab	5.07ab	5.18 ab	4.63b	0.16	< 0.05

*Note*: Means equal letters in the same row are not significantly different (*p* < 0.05).

Abbreviation: SEM, standard error of the mean.

^a^ Samples subjected only to 30 days of prior commercial vacuum aging.

^b^ Samples subjected to 30 days of prior commercial vacuum aging, followed by the experimental aging process, with or without lipid coating.

^c^ Structured hedonic scale (1—disliked extremely, to 7—liked extremely).

**FIGURE 1:**
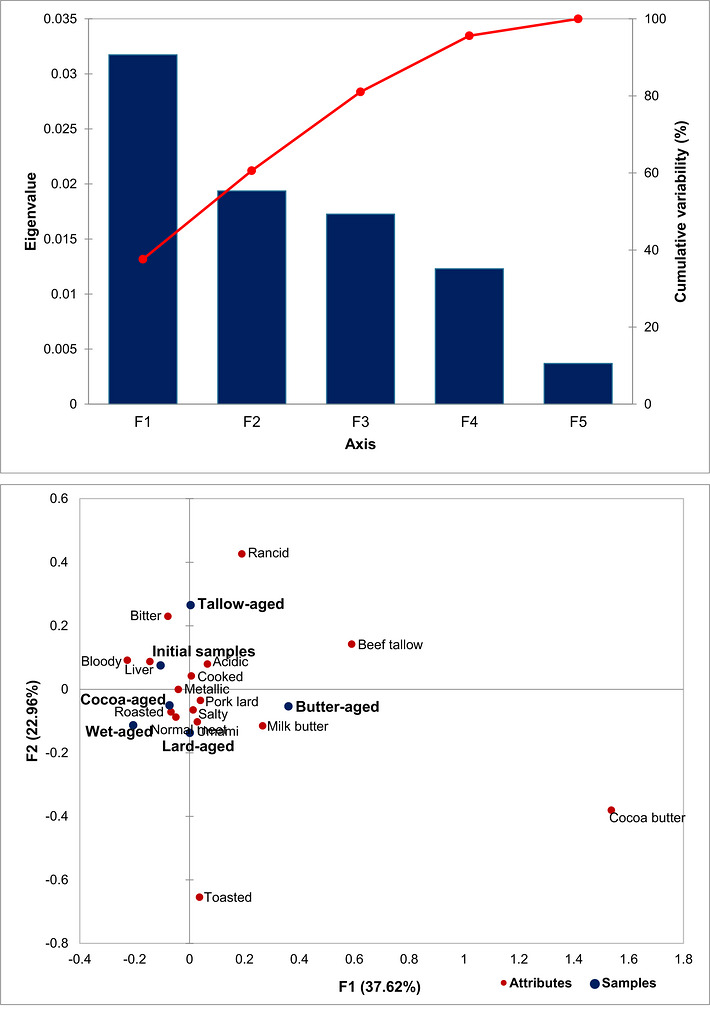
Eigenvalues for correspondence analysis (CA) and CA of the sensory profile and CATA method of beef‐aged with lipid coating. Initial samples were those subjected only to 30 days of prior commercial vacuum aging. Wet‐aged and lipid‐coated samples were those subjected to 30 days of prior commercial vacuum aging, followed by the experimental aging process, with or without lipid coating.

**FIGURE 2:**
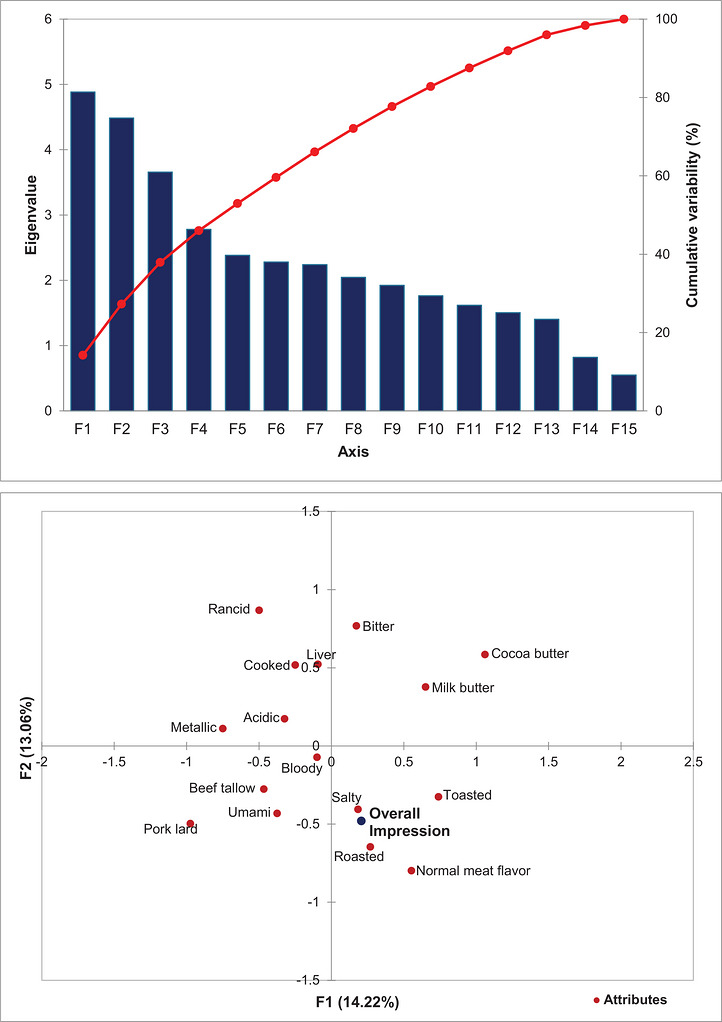
Eigenvalues for internal preference map (IPM) and IPM of beef‐aged with lipid coating.

The CATA method terms for different samples are illustrated in Figure 1 by CA, which explains 60.58% of the data variation (F1: 37.62% and F2: 22.96%), totaling 81.04% of the variation when using factor 3 (F3: 20.46%; Figure S1). The plot clearly represents how products were evaluated and associated with attributes. For example, butter‐aged samples were more associated with positive descriptors such as “milk butter flavor”; tallow‐aged samples were closer to descriptors such as “bitter” and “rancid”, whereas wet‐aged and cocoa‐ and lard‐aged samples were grouped together and associated with positive attributes like “normal”, “roasted”, and “umami”. Initial samples were linked to negative characteristics such as “liver” and “blood”, indicating a stronger association with these attributes compared with the other treatments. These sensory associations are consistent with the differences observed in hedonic scores, particularly the lower acceptance of tallow‐aged samples and the relatively higher scores for lard‐ and wet‐aged samples. Additionally, an IPM (Figure 2) was developed, associating overall impression results with CATA attributes, explaining 27.28% of the results' variation (14.22% in factor 1 and 13.06% in factor 2). Higher sensory preference was associated with attributes such as salty and umami flavors, as well as roasted and toasted flavors and the normal flavor of meat.

A total of 85 volatile compounds were qualitatively identified across treatments (Table 5), classified into different chemical groups: alcohols and esters were the most abundant (17 each), followed by alkanes (16), aldehydes (11), ketones (11), carboxylic acids (6), furans (3), ether (1), phenol (1), lactone (1), and sulfide (1). Initial samples contained only two unique compounds (one alkane and one ketone), while wet‐aged samples contained one acid, one alkane, one ester, and two aldehydes. Five compounds were exclusively identified in butter‐aged samples, including two alcohols, one alkane, one ester, and one phenol. A single alcohol was exclusively identified in lard‐aged samples, while tallow‐aged samples contained two unique alcohols. No unique compounds were found in cocoa‐aged samples. However, 40 additional compounds were identified across multiple treatments without specific grouping, while 30 compounds were common to all treatments, distributed among alkanes (7), esters (7), aldehydes (6), alcohols (4), ketones (4), acids (1), and furans (1).

**TABLE 5:** Volatile compounds identified in beef‐aged with lipid coating.

Tratament	Class	R.T. (min)	Compound	Initial samples^a^	Wet‐aged^b^	Butter‐aged^b^	Cocoa‐aged^b^	Lard‐aged^b^	Tallow‐aged^b^
Initial samples^a^	Alkane	18.028	2,3,4‐Trimethylpentane	x					
	Ketone	24.570	Heptan‐3‐one	x					
Wet‐aged^b^	Acid	42.742	Decanoic acid		x				
	Aldehyde	28.158	(E)‐hept‐2‐enal		x				
	Aldehyde	13.474	3‐Methylbutanal		x				
	Alkane	34.064	Undec‐1‐ene		x				
	Ester	32.606	3‐Methylbutyl butanoate		x				
Butter‐aged^b^	Alcohol	11.962	2‐Methylpropan‐1‐ol			x			
	Alcohol	31.925	Phenylmethanol			x			
	Alkane	48.797	Heptadec‐1‐ene			x			
	Ester	21.383	Methyl pentanoate			x			
	Phenol	28.734	Phenol			x			
Lard‐aged^b^	Alcohol	45.443	Dodecan‐1‐ol					x	
Tallow‐aged^b^	Alcohol	28.463	Heptan‐1‐ol						x
	Ester	18.477	2‐Methylpropyl ethanoate						x
All trataments	Acid	11.325	Ethanoic acid	x	x	x	x	x	x
	Alcohol	23.558	Hexan‐1‐ol	x	x	x	x	x	x
	Alcohol	29.008	Oct‐1‐en‐3‐ol	x	x	x	x	x	x
	Alcohol	31.212	2‐Ethylhexan‐1‐ol	x	x	x	x	x	x
	Alcohol	25.273	Nonane	x	x	x	x	x	x
	Aldehyde	28.912	Benzaldehyde	x	x	x	x	x	x
	Aldehyde	25.394	Heptanal	x	x	x	x	x	x
	Aldehyde	20.335	Hexanal	x	x	x	x	x	x
	Aldehyde	34.678	Nonanal	x	x	x	x	x	x
	Aldehyde	30.197	Octanal	x	x	x	x	x	x
	Aldehyde	15.238	Pentanal	x	x	x	x	x	x
	Alkane	29.984	Decane	x	x	x	x	x	x
	Alkane	38.396	Dodecane	x	x	x	x	x	x
	Alkane	49.053	Heptadecane	x	x	x	x	x	x
	Alkane	47.475	Hexadecane	x	x	x	x	x	x
	Alkane	43.862	Tetradecane	x	x	x	x	x	x
	Alkane	41.469	Tridecane	x	x	x	x	x	x
	Alkane	34.384	Undecane	x	x	x	x	x	x
	Ester	24.025	2‐Methylbutyl acetate	x	x	x	x	x	x
	Ester	23.925	3‐Methylbutyl ethanoate	x	x	x	x	x	x
	Alcohol	18.482	Pentan‐1‐ol	x	x	x	x	x	x
	Ester	37.788	Hex‐3‐enyl butanoate	x	x	x	x	x	x
	Ester	20.150	Ethyl butanoate	x	x	x	x	x	x
	Ester	16.307	Methyl butanoate	x	x	x	x	x	x
	Ester	11.536	Dimethyl carbonate	x	x	x	x	x	x
	Furan	14.049	2‐Methyloxolane	x	x	x	x	x	x
	Ketone	10.689	Butan‐2‐one	x	x	x	x	x	x
	Ketone	14.639	Pentan‐2‐one	x	x	x	x	x	x
	Ketone	15.104	Pentan‐3‐one	x	x	x	x	x	x
	Ketone	29.258	6‐Methylhept‐5‐en‐2‐one	x	x	x	x	x	x
Not grouped	Acid	18.276	Butanoic acid	x		x	x		
	Acid	27.847	Hexanoic acid	x		x	x	x	x
	Acid	40.008	Nonanoic acid	x	x	x		x	x
	Acid	23.192	Pentanoic acid				x		x
	Alcohol	33.058	Octan‐1‐ol	x				x	
	Alcohol	21.581	Pentan‐1‐ol				x		x
	Alcohol	48.823	Tetradecan‐1‐ol	x	x		x		x
	Alcohol	47.175	Tridecan‐1‐ol				x		
	Alcohol	19.705	Butane‐2,3‐diol				x		x
	Alcohol	19.633	(2R,3R)‐Butane‐2,3‐diol			x	x		x
	Alcohol	17.145	2‐Methylbutan‐1‐ol			x	x		x
	Alcohol	16.930	3‐Methylbutan‐1‐ol			x	x		
	Aldehyde	13.788	2‐Methylbutanal	x	x	x	x		x
	Aldehyde	38.756	Decanal	x	x			x	x
	Alkane	30.930	Dec‐1‐yne	x	x		x	x	
	Alkane	20.815	Oct‐2‐ene	x	x				
	Alkane	13.938	Cyclohexane	x			x		x
	Alkane	10.977	Hexane	x	x		x	x	x
	Alkane	45.844	Pentadecane	x	x		x	x	x
	Alkane	9.714	Pentane		x	x	x		x
	Ester	30.050	3‐Hexen‐1‐yl acetate	x			x	x	
	Ester	30.315	Hexyl ethanoate	x	x			x	x
	Ester	18.825	Methyl 2‐methylbutanoate	x		x	x		x
	Ester	32.388	3‐Methylbutyl butanoate	x	x			x	x
	Ester	34.592	3‐Methylbutyl 3‐methylbutanoate		x			x	
	Ester	21.584	Pentyl formate		x	x		x	
	Ester	29.697	Ethyl hexanoate	x	x	x		x	x
	Ester	12.063	Methyl propanoate	x		x	x	x	x
	Ester	15.763	Ethyl propanoate	x	x				x
	Ether	16.633	1‐Methoxypentane	x	x			x	
	Furan	36.405	4‐Hydroxyoxolan‐2‐one	x	x				x
	Furan	25.944	Furan‐2(5H)‐one	x	x				x
	Ketone	10.432	Butane‐2,3‐dione	x		x	x		
	Ketone	29.092	Octane‐2,3‐dione	x	x	x			
	Ketone	24.720	Heptan‐2‐one			x	x	x	x
	Ketone	17.803	2‐Methylpentan‐3‐one	x		x	x		
	Ketone	15.786	3‐Hydroxybutan‐2‐one	x		x	x		
	Ketone	13.448	3‐Methylbutan‐2‐one	x					x
	Lactone	26.001	γ‐Butyrolactone			x	x		x
	Sulphide	17.930	(methyldisulfanyl)methane			x	x		

Abbreviation: R.T., retention time in minutes.

^a^ Samples subjected only to 30 days of prior commercial vacuum aging.

^b^ Samples subjected to 30 days of prior commercial vacuum aging, followed by the experimental aging process, with or without lipid coating.

**FIGURE 3:**
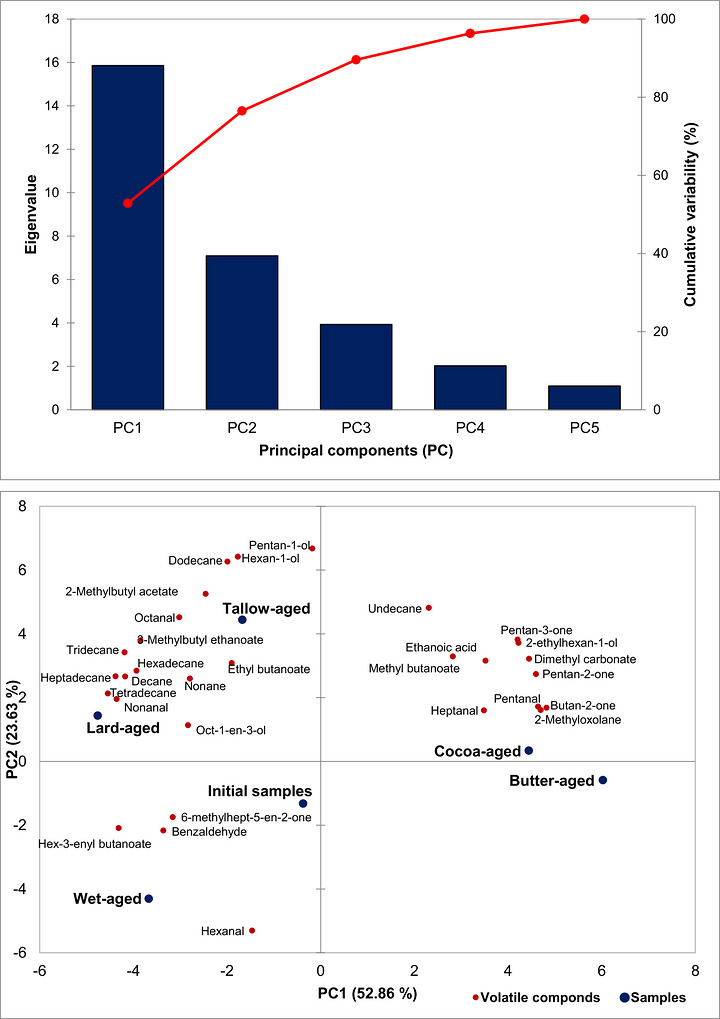
Eigenvalues for principal component analysis (PCA) and PCA score plot of volatile compounds in aged beef with lipid coating and cooked. Initial samples were those subjected only to 30 days of prior commercial vacuum aging. Wet‐aged and lipid‐coated samples were those subjected to 30 days of prior commercial vacuum aging, followed by the experimental aging process, with or without lipid coating.

To illustrate treatment variations based on individual volatile compounds, a PCA was created using the volatile compounds present in all treatments (Figure 3; Tables S1 and S2). The first principal component (PC1) explained 52.86% of the variation, while the second (PC2) explained 23.63%, totaling 76.49% of variance. Initial and wet‐aged samples were closely positioned in the lower quadrant near odor‐important compounds like hexanal and benzaldehyde. In the upper quadrant, tallow‐aged and lard‐aged samples were associated with compounds like octanal and nonanal. In contrast, butter‐aged and cocoa‐aged samples clustered near pentanal, butan‐2‐one, and 2‐methyloxolane.

To investigate the relationships among the evaluated variables, a Pearson's correlation analysis was performed (Table 6). Instrumental texture (SSF) did not show significant correlations with any of the other variables evaluated in this study. Among the significant and strong positive correlations (*r *= 0.70–0.90), notable associations were observed between sensory juiciness and tenderness (*r* = 0.72), flavor and overall impression (*r* = 0.85), as well as between the color parameters L* and hue angle (*r* = 0.74), and a* and b* (*r* = 0.82). Very strong positive correlations (*r* > 0.90) were observed between Chroma and a* (*r* = 0.96), and between Chroma and b* (*r* = 0.94).

**TABLE 6:** Pearson's correlation coefficients among physicochemical, sensory, and instrumental quality attributes of beef‐aged with lipid coating.

	Aw	CL	SSF	TD^a^	AR^a^	FL^a^	JC^a^	OI^a^	L* ^b^	a* ^b^	b* ^b^	C* ^b^
CL	0.61^*^											
SSF	0.22	−0.12										
TD^a^	0.10	−0.02	−0.13									
AR^a^	0.05	0.06	0.30	0.26								
FL^a^	0.12	0.24	−0.02	0.61^*^	0.60^*^							
JC^a^	0.44^*^	0.25	−0.14	0.72^*^	0.12	0.50^*^						
OI^a^	0.17	0.18	0.15	0.58^*^	0.62^*^	0.85^*^	0.56^*^					
L* ^b^	−0.53^*^	−0.27	−0.07	0.10	0.13	0.18	−0.21	0.08				
a* ^b^	0.69^*^	0.45^*^	0.27	−0.05	0.12	0.10	0.04	0.14	−0.02			
b* ^b^	0.45^*^	0.30	0.15	−0.06	0.05	0.12	−0.03	0.03	0.41^*^	0.82^*^		
C* ^b^	0.61^*^	0.39^*^	0.20	−0.07	0.06	0.08	0.01	0.06	0.18	0.96^*^	0.94^*^	
h* ^b^	−0.04	−0.04	−0.04	0.00	0.04	0.11	−0.10	−0.07	0.74^*^	0.29	0.77^*^	0.52^*^

*Note*: For correlation analysis, sensory scores were averaged across consumers within each steak sample so that all variables shared the same experimental unit (steak).

Abbreviations: a*, redness; AR, aroma; b*, yellowness; C*, chroma; FL, flavor; h*, hue angle; JC, juiciness; L*, lightness; OI, overall impression; SSF, slice shear force (N); TD, sensory tenderness.

* Significant correlations at *p *< 0.05.

^a^ To include sensory data in this correlation analysis, the average scores from the same panel of tasters for each steak were used.

^b^ Instrumental color data were based only on Day 0 of the display.

## 4 Discussion

The cracks observed in the lipid coating across treatments in this study were likely caused by the rapid crystallization of lipid crystals. A similar phenomenon was previously reported by Rezende‐de‐Souza et al. (2024), who evaluated the aging of beef loin for 28 days with butter and pork lard coatings. The occurrence of cracks in the lipid coatings may have influenced surface darkening through myoglobin oxidation, as these openings allowed direct oxygen penetration into the meat–lipid system. Oxygen exposure promotes the conversion of myoglobin into metmyoglobin, leading to a color shift from red to brown (King et al. 2023). Additionally, the use of twine to suspend the meat during aging may have further facilitated oxygen ingress. This could also have accelerated lipid oxidation, generating free radicals that contributed to color deterioration. In addition, the surface dehydration process may also have contributed to the darkening of the meat's surface, as is the case with dry‐aged meats (Bhat et al. 2018; Dransfield 1993).

The total loss observed in the samples during the aging process reflects dehydration that occurred mainly on the first day, when the lipid coating had not yet been applied. This initial dehydration step was intentionally included to improve lipid adhesion and therefore represents a controlled technological step rather than a continuous drying process. These losses were lower than those typically reported for conventional dry‐aged beef, where weight reduction can reach up to 50% (Bernardo et al. 2020; Bernardo, Silva, et al. 2020; Dashdorj et al. 2016). It is important to note that such high losses are generally associated with longer aging periods and specific processing conditions, such as extended exposure time, air velocity, and relative humidity, which differ from those applied in the present study.

The absence of packaging during dry aging not only promotes water loss but also reduces surface water activity; as the process progresses, surface water activity can approach 0.94 (Bernardo et al. 2020; Bernardo, Silva, et al. 2020). In the present study, most moisture loss likely occurred during the initial 24‐h period prior to lipid application, whereas the subsequent lipid coating may have acted as a physical barrier, limiting further water evaporation from the meat surface. For these samples, lipid‐coated samples had surface water activity values similar to those observed in the inner regions of dry‐aged meat reported in other studies (Bernardo, Silva, et al. 2020; Cônsolo et al. 2024; Ribeiro et al. 2021), suggesting that the coating helped maintain surface moisture at levels comparable to protected or internal muscle regions rather than exposed dry‐aged surfaces, despite slight variations between treatments.

Color measurements indicated that aging reduced redness, yellowness, and chroma compared to initial samples, reducing color saturation and increasing hue angle. These changes may be linked to reduced metmyoglobin reductase activity (MRA) during aged process. This is because the formation of metmyoglobin activates MRA, which, using nicotinamide adenine dinucleotide (NADH), reduces metmyoglobin (brown pigment) to oxymyoglobin (red pigment) (King et al. 2023). Over time, NADH depletion reduces MRA activity, leading to progressive darkening. In wet aging, this process is slower due to limited oxygen availability, but oxidation still occurs. This mechanism also may be explains the darkening observed during the storage time. From Day 4 onward, samples darkened more rapidly, with further intensification by Day 6.

Instrumental shear force values for all treatments were below 39 N, classifying them as “very tender” according to ASTM International guidelines (ASTM, F2925‐11 2011). Sensory evaluation confirmed this tenderness, with acceptance scores ≥ 5 (“liked” to “liked very much”). Aroma, flavor, and overall impression scores differed significantly among treatments, with lower scores observed for tallow‐aged samples. However, despite statistical significance, in general, subtle improvements in these sensory attributes were observed in some lipid‐coated aging treatments compared to initial and wet‐aged samples. Thus, the previously formulated hypothesis, reported in different non‐scientific dissemination channels (websites and social media) by meat producers regarding the transfer of flavor from the lipid used in aging coatings to the meat finds partial support in the present results, particularly for aroma, flavor, and overall impression.

The CATA method, combined with the profile of volatile compounds, contributed to suggesting possible variations in the sensory profile of the different samples. Initial and wet‐aged samples were associated with the flavor of cooked and roasted meat, as well as with the flavors of blood, liver, and metallic notes. These flavors are intensified during the heating of the meat, a process that favors the production of hexanal, a compound positively associated with beef but which can be undesirable when its concentration is too high (Calkins and Hodgen 2007; Kerth et al. 2023). Barker et al. (2023) identified a reduction in hexanal with prolonged vacuum aging of beef; at 28 days of aging, the standard error of the least‐squares mean was 80.87, decreasing to 54.94 at 35 days, and reaching 37.68 at 49 days, indicating that the closer to the slaughter day, the higher the hexanal concentration in the meat. Benzaldehyde, an aldehyde associated with an almond‐like flavor (Calkins and Hodgen 2007; Hicks et al. 2023), can resemble a bitter taste, and the bitter taste was grouped with initial and wet‐aged samples.

Hexadecane, tetradecane, heptadecane, hexan‐1‐ol, pentan‐1‐ol, and nonane are compounds associated with grass‐fed animals, resulting in flavors reminiscent of woody, grassy, fatty, and weak metallic notes (Calkins and Hodgen 2007). These compounds were grouped in lard‐aged and tallow‐aged samples, and this may help explain the lower sensory acceptance of tallow‐aged samples, particularly due to their association with rancid and bitter descriptors in the CATA analysis. Nonanal also contributes to herbaceous and earthy flavors and is associated with lipid oxidation processes (Calkins and Hodgen 2007; Dinh et al. 2025), being linked to lard‐aged and tallow‐aged samples. Additionally, tallow‐aged samples were more associated with the descriptor “rancid,” possibly due to their higher association with the aldehyde nonanal.

Acetic acid was grouped with butter‐aged and cocoa‐aged samples, and studies report an increase in the concentration of this acid with prolonged vacuum aging of beef, resulting in a final product with a more pronounced acidity (Barker et al. 2023; Mallick et al. 2021). Samples from these two lipid‐coated aging methods were associated with a sour taste when evaluated using the CATA method. In addition to ethanoic acid, other compounds may have also influenced this perception, such as heptanal and 2‐ethylhexan‐1‐ol. These compounds have been previously associated with woody, floral, grassy, fatty, citrus, sour, fruity, and menthol flavors and are naturally present in beef as primary products of lipid oxidation and as results of the Maillard reaction (Calkins and Hodgen 2007; Dinh et al. 2025; Sohail et al. 2022). Ketones butan‐2‐one and pentan‐2‐one, along with the furan 2‐methyloxolone, were grouped in butter‐aged and cocoa‐aged samples and are precursor compounds of a positive sweet flavor in cooked meat, widely reported in beef, pork, and crab (Sohail et al. 2022), which may partially explain the relatively higher aroma and flavor scores observed for these treatments compared to tallow‐aged samples.

Samples with higher overall impression scores were farther from those with flavors such as rancid, liver, metallic, bitter, and sour, which were particularly characteristic of samples aged with lipid coatings. Thus, in summary, the type of coating used in the aging process impacts the formation of the volatile profile of beef. The combination of lipid coatings such as butter, cocoa butter, lard, pork, and beef tallow results in different profiles of volatile compounds, which in turn influence the aroma, flavor, and overall impression of the meat, reflecting consumer preferences and the sensory characteristics of the samples. However, a sensory method with trained panelists may be more efficient in detecting flavor variation in meats aged with different lipid coatings.

The absence of significant correlations between instrumental tenderness (SSF) and sensory tenderness suggests that the slight variations in shear force among treatments were insufficient to be perceived by consumers. However, the strong association between juiciness and sensory tenderness suggests that samples perceived as juicier were also perceived as more tender, which may be related to lubrication effects during mastication (Savell and Cross 1988). The strong correlation between flavor and overall impression reinforces that flavor was one of the main drivers of consumer acceptance in the present study. Thus, even though lipid‐coated aging did not markedly alter instrumental quality parameters, subtle modifications in flavor‐related attributes appeared sufficient to influence consumer perception.

The positive association between water activity with redness and chroma suggests that samples with lower surface dehydration exhibited more vivid color characteristics. Conversely, the negative correlation between water activity and lightness may indicate that lower surface dehydration contributed to darker and more saturated meat color. This behavior supports the discussion presented earlier regarding the role of lipid coatings in reducing superficial dehydration during aging.

Overall, the correlation analysis reinforces that sensory perception in lipid‐coated aged beef was more closely associated with characteristics related to flavor and appearance than with instrumental tenderness. Moreover, these relationships complement the PCA of volatile compound, which indicated associations between specific volatile profiles and sensory descriptors linked to consumer acceptance.

## 5 Conclusion

Beef‐aged with different lipid coatings showed physicochemical characteristics comparable to those of initial samples and wet‐aged samples. Sensory evaluation revealed significant differences among treatments for aroma, flavor, and overall impression. Tallow‐aged samples showed a greater association with descriptors less commonly related to positive meat attributes. In contrast, butter‐ and lard‐aged samples were more frequently associated with desirable attributes such as roasted, umami, and typical meat flavor. Lipid‐coated aging influenced the volatile profile of the samples, contributing to the differentiation of sensory attributes observed in both hedonic evaluation and CATA analysis. Despite these effects, lipid coatings did not provide substantial advantages over conventional wet aging in terms of overall palatability. Additionally, the use of lipid coatings may contribute to reduced moisture loss compared to conventional dry‐aging processes reported in the literature. Overall, lipid‐coated aging can be considered a complementary approach for flavor modulation rather than a replacement for conventional aging methods. Among the evaluated treatments, pork lard may represent a cost‐effective alternative to butter, without negatively affecting the sensory quality of the final product under the conditions evaluated in this study.

## Author Contributions

**Jonatã Henrique Rezende‐de‐Souza**: conceptualization, data curation, formal analysis, investigation, methodology, validation, visualization, writing – original draft, writing – review and editing, project administration. **Kathelen Lethicia Cavalheri Rodrigues Jacinto**: investigation, writing – review and editing, visualization. **Isaac de Lima Vieira**: investigation, writing – review and editing. **Gabriela Lima de Oliveira**: investigation, writing – review and editing. **Isabela Benfica de Barros**: investigation, writing – review and editing. **Flavio Andre Bolini Cardello**: investigation. **Dyana Carla Lima Hargreaves Noguera**: investigation. **Vanessa Cristina Francisco**: investigation, writing – review and editing, formal analysis. **Renata Tieko Nassu**: resources, writing – review and editing. **Helena Maria Andre Bolini**: writing – review and editing, formal analysis. **Sérgio Bertelli Pflanzer**: conceptualization, methodology, funding acquisition, resources, project administration, supervision, writing – review and editing, validation.

## Conflicts of Interest

The authors declare that they have no conflicts of interest.

## Supporting information




**Supplementary Figure 1**: jfds71240‐sup‐0001‐FigureS1.docx

**Supplementary Table 1**: jfds71240‐sup‐0002‐TableS1.docx

**Supplementary Table 2**: jfds71240‐sup‐0003‐TableS2.docx
