# Supplementary material for: From Yield to Flavor: The Role of Lipid Coatings in Beef Aging
Source: J Food Sci. 2026 Jun 29;91(7):e71240. doi: 10.1111/1750-3841.71240 (PMC13312044; doi:10.1111/1750-3841.71240)
Supplement: Supplementary file 1 — Supplementary Figure 1: jfds71240‐sup‐0001‐FigureS1.docx [file JFDS-91-0-s001.docx]

**SUPPLEMENTARY FIGURE 1.** Correspondence analysis (F1 × F3, and F2 × F3) for CATA analysis of beef aged with lipid coating. Initial samples were those subjected only to 30 days of prior commercial vacuum aging. Wet-aged and lipid-coated samples were those subjected to 30 days of prior commercial vacuum aging, followed by the experimental aging process, with or without lipid coating.
