# Supplementary material for: From Yield to Flavor: The Role of Lipid Coatings in Beef Aging
Source: J Food Sci. 2026 Jun 29;91(7):e71240. doi: 10.1111/1750-3841.71240 (PMC13312044; doi:10.1111/1750-3841.71240)
Supplement: Supplementary file 2 — Supplementary Table 1: jfds71240‐sup‐0002‐TableS1.docx [file JFDS-91-0-s002.docx]

**SUPPLEMENTARY TABLE 1.** Correlations between volatile compounds and principal components (PC).

| **Volatile compounds** | **PC1** | **PC2** | **PC3** | **PC4** | **PC5** |
| --- | --- | --- | --- | --- | --- |
| Butan-2-one | 0.938 | 0.232 | -0.004 | 0.141 | -0.217 |
| Ethanoic acid | 0.570 | 0.445 | -0.159 | 0.147 | 0.656 |
| Dimethyl carbonate | 0.898 | 0.435 | -0.013 | -0.041 | -0.046 |
| 2-Methyloxolane | 0.949 | 0.218 | -0.133 | 0.166 | 0.084 |
| Pentan-2-one | 0.928 | 0.370 | -0.001 | 0.003 | -0.039 |
| Pentan-3-one | 0.849 | 0.516 | -0.010 | -0.111 | 0.025 |
| Pentanal | 0.974 | 0.227 | -0.004 | -0.014 | 0.010 |
| Methyl butanoate | 0.853 | 0.502 | -0.035 | -0.133 | 0.039 |
| Pentan-1-ol | -0.036 | 0.901 | -0.418 | 0.109 | -0.005 |
| Ethyl butanoate | -0.884 | 0.360 | 0.195 | -0.223 | 0.009 |
| Hexanal | -0.296 | -0.716 | -0.617 | -0.135 | -0.032 |
| Hexan-1-ol | -0.357 | 0.867 | -0.297 | 0.175 | -0.049 |
| 3-Methylbutyl acetate | -0.777 | 0.513 | -0.104 | -0.178 | 0.302 |
| 2-Methylbutyl acetate | -0.496 | 0.709 | -0.488 | -0.111 | 0.012 |
| Nonane | -0.880 | 0.264 | 0.171 | -0.356 | -0.027 |
| Heptanal | 0.704 | 0.216 | 0.581 | -0.215 | 0.272 |
| Benzaldehyde | -0.678 | -0.292 | -0.094 | 0.658 | 0.110 |
| Oct-1-en-3-ol | -0.572 | 0.153 | 0.679 | 0.396 | 0.176 |
| 6-methylhept-5-en-2-one | -0.638 | -0.236 | -0.419 | 0.561 | 0.217 |
| Decane | -0.845 | 0.462 | 0.221 | 0.011 | -0.152 |
| Octanal | -0.384 | 0.417 | 0.786 | -0.238 | 0.065 |
| 2-ethylhexan-1-ol | 0.711 | 0.426 | 0.125 | 0.465 | -0.285 |
| Undecane | 0.466 | 0.650 | 0.276 | 0.482 | -0.227 |
| Nonanal | -0.564 | 0.351 | 0.747 | -0.016 | -0.028 |
| Hex-3-enyl butanoate | -0.871 | -0.282 | 0.268 | 0.299 | 0.023 |
| Dodecane | -0.402 | 0.846 | -0.325 | 0.082 | 0.100 |
| Tridecane | -0.610 | 0.610 | -0.468 | -0.191 | 0.016 |
| Tetradecane | -0.918 | 0.288 | -0.267 | -0.058 | 0.009 |
| Hexadecane | -0.794 | 0.384 | -0.211 | -0.060 | -0.417 |
| Heptadecane | -0.843 | 0.359 | 0.335 | 0.218 | -0.021 |
