# Supplementary material for: From Yield to Flavor: The Role of Lipid Coatings in Beef Aging
Source: J Food Sci. 2026 Jun 29;91(7):e71240. doi: 10.1111/1750-3841.71240 (PMC13312044; doi:10.1111/1750-3841.71240)
Supplement: Supplementary file 3 — Supplementary Table 2: jfds71240‐sup‐0003‐TableS2.docx [file JFDS-91-0-s003.docx]

**SUPPLEMENTARY TABLE 2.** Contribution of the volatile compounds and treatments with each principal component (PC).

| **Volatile compounds** | **PC1** | **PC2** | **PC3** | **PC4** | **PC5** |
| --- | --- | --- | --- | --- | --- |
| Butan-2-one | 5.544 | 0.761 | 0.000 | 0.981 | 4.291 |
| Ethanoic acid | 2.048 | 2.788 | 0.647 | 1.062 | 39.273 |
| Dimethyl carbonate | 5.089 | 2.665 | 0.004 | 0.083 | 0.195 |
| 2-Methyloxolane | 5.677 | 0.670 | 0.450 | 1.358 | 0.644 |
| Pentan-2-one | 5.435 | 1.928 | 0.000 | 0.000 | 0.136 |
| Pentan-3-one | 4.547 | 3.751 | 0.002 | 0.605 | 0.056 |
| Pentanal | 5.978 | 0.730 | 0.001 | 0.010 | 0.009 |
| Methyl butanoate | 4.587 | 3.556 | 0.031 | 0.876 | 0.141 |
| Pentan-1-ol | 0.008 | 11.456 | 4.444 | 0.587 | 0.002 |
| Ethyl butanoate | 4.932 | 1.833 | 0.967 | 2.458 | 0.008 |
| Hexanal | 0.552 | 7.230 | 9.687 | 0.903 | 0.095 |
| Hexan-1-ol | 0.804 | 10.592 | 2.252 | 1.513 | 0.218 |
| 3-Methylbutyl acetate | 3.808 | 3.709 | 0.275 | 1.555 | 8.293 |
| 2-Methylbutyl acetate | 1.551 | 7.098 | 6.063 | 0.613 | 0.014 |
| Nonane | 4.878 | 0.986 | 0.742 | 6.250 | 0.067 |
| Heptanal | 3.122 | 0.660 | 8.593 | 2.282 | 6.770 |
| Benzaldehyde | 2.902 | 1.206 | 0.225 | 21.382 | 1.100 |
| Oct-1-en-3-ol | 2.063 | 0.332 | 11.736 | 7.756 | 2.828 |
| 6-methylhept-5-en-2-one | 2.565 | 0.784 | 4.473 | 15.538 | 4.285 |
| Decane | 4.507 | 3.005 | 1.246 | 0.005 | 2.116 |
| Octanal | 0.928 | 2.451 | 15.732 | 2.801 | 0.384 |
| 2-ethylhexan-1-ol | 3.186 | 2.566 | 0.399 | 10.662 | 7.404 |
| Undecane | 1.370 | 5.962 | 1.940 | 11.463 | 4.692 |
| Nonanal | 2.004 | 1.738 | 14.200 | 0.013 | 0.070 |
| Hex-3-enyl butanoate | 4.783 | 1.121 | 1.832 | 4.417 | 0.047 |
| Dodecane | 1.019 | 10.096 | 2.692 | 0.334 | 0.920 |
| Tridecane | 2.344 | 5.256 | 5.568 | 1.808 | 0.023 |
| Tetradecane | 5.313 | 1.168 | 1.810 | 0.169 | 0.007 |
| Hexadecane | 3.973 | 2.082 | 1.136 | 0.179 | 15.874 |
| Heptadecane | 4.483 | 1.821 | 2.856 | 2.336 | 0.041 |
| Total (%) | 100 | 100 | 100 | 100 | 100 |
| **Treatments** | **PC1** | **PC2** | **PC3** | **PC4** | **PC5** |
| Initial samples | 0.147 | 4.108 | 0.112 | 73.384 | 5.583 |
| Wet-aged | 14.151 | 43.582 | 11.888 | 11.669 | 2.043 |
| Butter-aged | 38.188 | 0.810 | 0.718 | 11.623 | 31.994 |
| Cocoa-aged | 20.771 | 0.267 | 2.279 | 0.325 | 59.691 |
| Lard-aged | 23.808 | 4.841 | 51.738 | 2.433 | 0.513 |
| Tallow-aged | 2.935 | 46.392 | 33.265 | 0.565 | 0.176 |
| Total (%) | 100 | 100 | 100 | 100 | 100 |
